# Anesthetic and obstetric outcomes in pregnant women undergoing cesarean delivery according to body mass index: Retrospective analysis of a single-center experience

**DOI:** 10.1016/j.amsu.2018.10.023

**Published:** 2018-11-02

**Authors:** Efrain Riveros-Perez, Jacob McClendon, Jennifer Xiong, Thomas Cheriyan, Alexander Rocuts

**Affiliations:** a Department of Anesthesiology and Perioperative Medicine, Medical College of Georgia at Augusta University, 1120 15th Street, Augusta, GA, 30912, USA; b Outcomes Research Consortium, Cleveland Clinic, OH, USA; c Lincoln Memorial University, Harrogate, TN, USA; d Medical College of Georgia at Augusta University, USA; e Department of Anesthesiology and Perioperative Medicine, Medical College of Georgia at Augusta University, USA

**Keywords:** Morbid obesity, Pregnancy, Anesthesia, Body mass index, Maternal outcomes

## Abstract

**Aim:**

To evaluate maternal, neonatal and anesthetic outcomes according to BMI in women undergoing cesarean section.

**Background:**

Increased incidence rates of obesity and morbid obesity have been reported in the United States. Pregnant obese patients are at increased risk of maternal and fetal complications, and obstetric and anesthetic management of these patients is especially challenging.

**Methods:**

A retrospective chart review of patients who underwent cesarean section in a single center between 2015 and 2016 was conducted. Anesthetic, obstetric and neonatal outcomes were analyzed in relation to levels of BMI.

**Results:**

Seven hundred and seventy one patients underwent cesarean section during the study period. The number of patients with normal BMI, obesity and morbid obesity was 213 (27.6%), 365 (47.3%) and 193 (25%), respectively. Sixty-one percent of the patients in morbidly obese group had at least one comorbidity (p < 0.01). We found no significant differences with respect to perioperative obstetric complications. Intraoperative blood loss was significantly higher in the morbidly obese group.

**Conclusion:**

Increasing BMI is associated with comorbidities such as hypertension and diabetes mellitus, and with increased intraoperative blood loss. We were unable to detect differences in other obstetric, anesthetic and neonatal outcomes.

## Highlights

• Incidence of morbid obesity is high in pregnancy.
• Morbid obesity is associated with medical and obstetric morbidities.
• Body mass index is associated with hypertension and diabetes mellitus in pregnant patients.
• Increased blood loss in cesarean section is associated with high body mass index.
• No significant maternal or anesthetic complications can be attributed solely to increasing body mass index.

## 1 Background

Obesity is a significant public health problem with increasing incidence in both the developed and the developing world. Body mass index (BMI) has increased by 0.4% worldwide over the last thirty years [1], and in the United States one in three adults have a BMI greater than 30 kg/m^2^ [2]. Data from the Behavioral Risk Factor Surveillance System revealed that morbid obesity, defined as the presence of a BMI >40 kg/m^2^ [3], doubled between 1986 and 2000 [4]. Morbidly obese individuals are at high risk for the coexistence of several comorbid conditions that affect different organ systems. Risk of developing coronary artery disease is increased by 50% and likelihood of developing atrial fibrillation is significantly higher in morbidly obese patients [5]. Risk of developing Diabetes Mellitus increases by 20% per 1 kg/m^2^ increase in BMI [6]. Other complications associated with morbid obesity include obstructive sleep apnea, gastroesophageal reflux and cerebrovascular disease [[7], [8], [9]].

Morbid obesity is present in 8% of women in reproductive age, and its incidence is increasing in pregnancy [2,10,11]. Excessive gestational weight gain and obesity during gestation are independent risk factors for maternal and fetal complications [12]. In addition to early-pregnancy complications such as increased risk of recurrent miscarriages and congenital anomalies, morbid obesity is associated with problems during late gestation that are relevant to the anesthesiologist. Morbidly obese patients are at increased risk for gestational hypertension, preeclampsia and gestational diabetes [13,14]. On the other hand, morbid obesity increases the risk of cesarean section and peripartum hemorrhage [15]. In addition, neonatal outcomes such as preterm birth and large for gestational age babies are more common in morbidly obese parturients [15].

As the incidence of morbid obesity is increasing in both the general and the obstetric population, the anesthesiologist must be prepared to customize a perioperative plan to take care of these patients in the labor and delivery wards and operating rooms. Hood and Dewan prospectively evaluated 117 morbidly obese parturients with matched controls, showing a higher incidence of epidural failure rate, obstetric complication rates and cesarean section [16]. Tonidandel et al. confirmed these findings, and also evidenced an increased risk of antenatal complications and prolonged first stage of labor [17]. Increasing BMI is a predictor of difficult placement and time to detect failure of labor epidural analgesia [18]. Regional anesthesia is recommended for obese patients in the obstetric setting, and an epidural catheter should be placed early in labor given the higher incidence of unplanned cesarean section in this population [19]. On the other hand, the risk for difficult or failed intubation is exceedingly high in morbidly obese pregnant patients [20]. Hemodynamic changes during anesthesia are also more prevalent in the obese parturient [21].

Since obesity has been strongly associated with obstetric, neonatal and anesthetic complications, and scarce reports have evaluated anesthetic and obstetric outcomes after cesarean delivery in this high-risk population; we retrospectively analyzed anesthetic, obstetric and neonatal outcomes in pregnant patients with different BMI, who underwent cesarean delivery at our institution between 2015 and 2016. We tested the hypothesis that obstetric, anesthetic and neonatal complications in morbidly obese pregnant patients undergoing cesarean delivery are associated with the degree of obesity measured by BMI.

## 2 Methods

This is an observational retrospective study based on chart review. The study is registered with ISRCTN registry (registration number: ISRCTN 16386326). After approval by the Institutional Review Board, a retrospective cohort study was conducted at a tertiary medical center. We included pregnant patients older than 18 years of age, who underwent cesarean section between January 2015 and January 2016. Patients corresponded to Robson groups 2, 4, 5, 6, 7, 8 and 9 [22]. Prenatal and outcome variables were obtained from the health documentation system of Augusta University. For analysis purposes, the patients were divided into three groups based on body mass index measured at admission: Non-obese patients (BMI <30 kg/m^2^), obese patients (BMI 30–39.9 kg/m^2^) and morbidly obese patients (BMI ≥ 40 kg/m^2^). We collected demographic variables: maternal age and ASA status, body mass index, maternal comorbidities, and information related to pregnancy: gestational age, parity, prior cesarean deliveries, indication for cesarean section, and obstetric comorbidities. We also recorded obstetric complications (bleeding requiring transfusion, infection, deep venous thrombosis/pulmonary embolism), wound infection, maternal disposition, total length of stay and maternal mortality. Apgar scores and birth weight was also recorded. Finally, anesthetic variables including anesthetic technique, failed neuraxial block, total intraoperative phenylephrine dose and anesthesia-related complications (difficult airway, wet tap, postdural puncture headache) were documented. Occurrence of wound infection was evaluated within 30 days after surgery by surgical notes. We compared the outcome variables between the three BMI groups described above. The study is reported in line with the STROCSS criteria [23].

Statistical analysis was performed using SPSS 20.0 (IBM, New York). Continuous data is presented as mean and standard deviation, and categorical data presented as frequency. Analysis of Variance (ANOVA) and *t*-test were used to compare categorical data and continuous data. Fisher's *t*-test was used for post hoc analysis. Kruskal-Wallis test was utilized for non-parametric variables using ranks. P < 0.05 was considered statistically significant. The Shapiro-Wilk test was used to check for normality of data.

## 3 Results

**Fig. 1:**
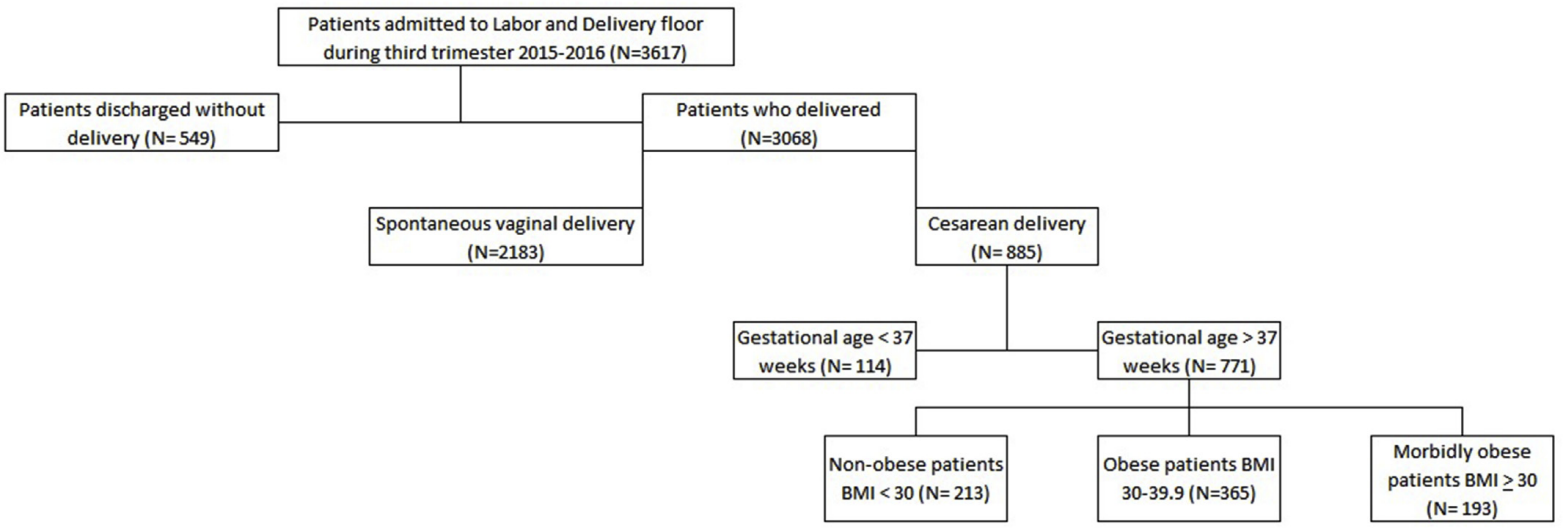
Flow Chart for patients who delivered during the study period.

Seven hundred and seventy one patients meeting our inclusion criteria underwent cesarean section during the study period, representing 29% of total deliveries. The number of patients labeled as normal BMI, obese and morbidly obese was 213 (27.6%), 365 (47.3%) and 193 (25%), respectively (Fig. 1). Mean age was slightly lower among patients who had normal BMI (27.09 ± 6.08) when compared to the obese (27.98 ± 5.9) and the morbidly obese (28.6 ± 5.8) groups, p = 0.037. Among the groups, the morbidly obese patients had the highest proportion of African American ethnicity (66%), p < 0.001. Gestational age at birth was marginally lower in the normal BMI group (36.2 ± 4 weeks) when compared to the morbidly obese (37 ± 3.7) and obese (37 ± 3.6) cohorts; p = 0.025. Sixty-one percent of the patients in morbidly obese group had at least one comorbidity (p < 0.01), with 26% having hypertension (p < 0.001), and 10.4% having diabetes mellitus (p = 0.002). There were no differences in the incidence of other comorbidities among the three groups (Table 1).

**Table 1:** Demographic variables between normal, obese and morbidly obese patients.

Characteristics	Normal (n = 213)	Obese (n = 365)	Morbidly Obese (n = 193)	*P*-value
Age (years)				
Median ± SD	27.05 ± 6.1	27.98 ± 5.9	28.9 ± 5.1	**0.036**
Gestational age at delivery (weeks)				
Median ± SD	35.7 ± 4.0	37.1 ± 3.1	36.7 ± 3.9	.023
Race, n (%)				
Asian	9 (4.2)	12 (3.3)	1 (0.5)	<**.001**
Black	95 (44.6)	209 (57.3)	128 (66.3)	
Caucasian	96 (45.1)	115 (31.5)	56 (29)	
Hispanic	9 (4.2)	28 (7.7)	5 (2.6)	
Other	4 (1.9)	1 (0.3)	3 (1.6)	
Gravidity				0.75
Parity				0.43
Medical comorbidities, n (%)	77 (41)	150 (44.8)	114 (61.3)	<**0.001**
Hypertension, n (%)	12 (5.7)	44 (12.1)	50 (25.9)	<**0.001**
Diabetes, n (%)	4 (1.9)	24 (6.6)	20 (10.4)	**.002**
Asthma, n (%)	10 (4.7)	22 (6)	18 (9.3)	.148
GERD, n (%)	10 (4.7)	22 (6)	14 (7.3)	.553
Anemia, n (%)	7 (3.3)	7 (1.9)	4 (2.1)	.554
Infectious diseases including HIV, Hepatitis B, Hepatitis C, HSV, n (%)	7 (3.3)	9 (2.5)	1 (0.5)	.148
Idiopathic thrombocytopenia, n (%)	6 (2.8)	4 (1.1)	2 (1)	.217
Smoking, n (%)	16 (7.5)	20 (5.5)	7 (3.6)	.233
Substance abuse, n (%)	13 (6.1)	13 (3.6)	1 (0.5)	**.009**

Data are reported as number with percent in parenthesis unless otherwise indicated. Abbreviations: SD, standard deviation. HIV, human immunodeficiency virus. HSV, herpes simplex virus. Analysis of Variance (ANOVA) and *t*-test were used to compare categorical data and continuous data. Fisher's *t*-test was used for post hoc analysis. P < 0.05 was considered statistically significant.

We found no significant differences with respect to perioperative obstetric complications. Intraoperative blood loss (estimated by quantification of collected blood and common surgical gauze per institutional protocol) was significantly higher in the morbidly obese group. However, the number of patients requiring blood transfusion was not statistically different among the groups. There was no difference in number of patients requiring emergent cesarean section, maternal disposition to the intensive care unit, postoperative infection, and postpartum hemorrhage. Sixty-seven percent of patients in the morbidly obese group were classified as American Society of Anesthesiologists physical status (ASA 3). No significant difference was observed between the groups with respect to anesthesia technique (neuraxial versus general), incidence of failed neuraxial block, phenylephrine dose or length of stay. There was no difference in neonatal outcome with respect to Apgar score at 1 and 5 min (Table 2). The post-hoc analysis confirmed intergroup significant differences for the positive findings (Table 3).

**Table 2:** Obstetric, anesthetic and neonatal outcomes.

Outcomes	Normal (n = 213)	Obese (n = 365)	Morbidly Obese (n = 193)	*P*-value
Blood loss (mL)				
Mean ± SD	719 ± 215	754 ± 365	816 ± 257	< **0.001**
Blood transfusion, units (%)	14 (6.6)	16 (4.4)	8 (4.1)	.425
Obstetric timing				.335
Emergent, n (%)	93 (43.7)	134 (36.7)	84 (43.5)	.151
Maternal disposition- Intensive Care Unit, n (%)	2 (0.9)	0 (0)	3 (1.6)	.077
Postoperative infection, n (%)	16 (7.5)	21 (5.8)	16 (8.3)	.483
Maternal mortality, n	0	0	0	
Neuraxial anesthesia, n (%)	179 (84)	326 (89.3)	166 (86.5)	.178
Physical status, n (%)				
ASA 1	13 (6.1)	10 (2.7)	3 (1.6)	<**0.001**
ASA 2	147 (69)	203 (55.6)	55 (28.5)	
ASA 3	49 (23)	149 (40.8)	129 (66.8)	
ASA 4	3 (1.4)	1 (0.3)	5 (2.6)	
ASA 5	0 (0)	1 (0.3)	0 (0)	
Failed neuraxial block, n (%)	6 (3)	20 (5.6)	8 (4.3)	.340
Phenylephrine dose (micrograms), n	249.25	263.40	262.10	.806
Post-dural puncture headache, n (%)	0 (0)	2 (0.6)	1 (0.6)	.562
Tongue-to-pharynx score, n (%)				
Mallampati 1	101 (47.9)	94 (26)	34 (17.8)	<**0.001**
Mallampati 2	86 (40.8)	192 (53,2)	75 (39.3)	
Mallampati 3	19 (9)	66 (18.3)	74 (38.7)	
Mallampati 4	5 (2.4)	9 (2.5)	8 (4.2)	
Apgar score at 5 min, n (%)				
< 7	42 (15)	44 (12.1)	24 (13)	0.835
7 - 10	181 (85)	321 (87.9)	168 (87.0)	
Length of Stay (days)	3.46	3.52	3.65	.832

Data are reported as number with percent in parenthesis unless otherwise indicated. Abbreviations: SD, standard deviation. ASA, American Society of Anesthesiologists physical status. SD, standard deviation. Analysis of Variance (ANOVA) and *t*-test were used to compare categorical data and continuous data. Fisher's *t*-test was used for post hoc analysis. P < 0.05 was considered statistically significant.

**Table 3:** Post-hoc analysis for intergroup differences in comorbidities.

Group		Group Comparisons				
	HTN	Non-obese	Obese	DM	Non-obese	Obese
Non-obese (n = 213)	12 (5.7)			4 (1.9)		
Obese (n = 365)	44 (12.1)	<.001		24 (6.6)	<.001	
Morbidly obese (n = 193)	50 (25.9)	<.001	.002	20 (10.4)	<.001	.02

Data are reported as number with percent in parenthesis unless otherwise indicated. Abbreviations: HTN, arterial hypertension. DM, diabetes mellitus.

## 4 Discussion

This study shows that at our institution we have a significant prevalence of obesity and morbid obesity in patients undergoing cesarean section. Our results also show that obesity and morbid obesity are associated with a higher prevalence of medical comorbidities such as hypertension and diabetes mellitus; however, we did not find differences with respect to obstetric complications comparing different levels of BMI. Although intraoperative bleeding increases with BMI, no differences in blood transfusion was noted. We did not find differences in rate of emergent cesarean section, postoperative bleeding or infection. High BMI was associated with higher ASA scores but there were no differences in major anesthetic outcomes. No differences in Apgar scores was found in relation to BMI.

The State of Georgia has experienced an increase in adult obesity rate from 20.6% in 2000 to 30.7% in 2016 [24]. In our institution, 72% of patients were labeled as obese or morbidly obese, reflecting the high risk posed by obesity during pregnancy in our population. We identified a higher prevalence of morbid obesity in patients with African American ethnicity. Our results are in line with the reported incidence of obesity in African American patients [25]. Black patients are susceptible to psychosocial, environmental and cultural factors that promote weight gain [26]. Several studies have documented an increased incidence of adverse pregnancy outcomes in morbidly obese patients, including hypertensive disorders and gestational diabetes mellitus [27]. In our study, 61% of patients presented with at least one comorbidity, mainly hypertensive disorders of pregnancy and diabetes mellitus, confirming the findings of other authors. We did not find difference in rate of preterm labor between groups; however, we found a marginal lower gestational age at delivery in normal BMI patients. These results confirm the findings reported by Kumari et al. [28]. On the other hand, Tonidandel et al. were not able to find differences in gestational age at birth between obese and non-obese parturients [17]. It is possible that obstetrical efforts to minimize the rate of delivery before term by cesarean section in obese patients resulted in a higher gestational age in this group of patients [29].

Conflicting reports regarding intraoperative blood loss in obese patients undergoing surgery in different settings have been documented. Bowditch et al. reported that obese patients having hip replacement surgery bled more compared to patients with normal BMI [30], whereas Wang et al. showed that obesity is a protective factor against the need to receive blood transfusion during coronary artery bypass surgery [31]. Our study showed that, although blood loss was higher in obese patients, no difference in transfusion rate occurred. An et al. also showed increased bleeding in high BMI patients undergoing cesarean section, with no mention of transfusion rates [32]. We might argue that obese patients tend to have significantly higher blood loss compared to non-obese patients having cesarean section, due to prolonged surgical times, difficult surgical access and dissection of tissues; however, these variables were not measured in our study. On the other hand, the fact that transfusion rates did not differ between groups in our study, might reflect our conservative transfusion therapies. Although the risk of transfusion in association with cesarean section is low, low baseline hemoglobin levels and obstetric conditions such as placenta previa, increase these risks [33]. We recommend optimization of hematocrit during the antenatal period in obese patients, to reduce the need for perioperative/peripartum transfusion.

We did not find an increase rate of postoperative infections in patients with high BMI. Conner et al. demonstrated a dose-response relationship between increasing BMI and risk of post-cesarean wound complications [34]. Myles et al. showed that obesity is an independent risk factor for post-cesarean infectious complications, even in an elective setting [35]. The inability of our study to detect these differences in infectious complications could be the result of a small sample. The effect of an insufficient sample can also explain that our study did not find difference in neonatal scores between obese and non-obese parturients. Chen et al. retrospectively analyzed 58.089 pregnant patients to evaluate the effect of BMI on Apgar scores. The authors found that maternal obesity is associated with significantly increased risk for low Apgar scores at birth [36].

Regarding the anesthetic technique, our results showed no difference in type of anesthesia, incidence of failed neuraxial block, need for ICU admission, and hemodynamic stability measured by the total dose of intraoperative phenylephrine. An et al. showed that ICU admission is the same regardless of BMI after cesarean section [32]. This is despite the increased blood loss and comorbidities in morbidly obese patients. These results might indicate the significant physiologic reserve that young pregnant patients have in general. As expected from current practice standards in the United States, the majority of our patient population had anesthesia for cesarean section under neuraxial blocks. Although difficulty in placing a neuraxial block in an obese patient might be expected to be difficult, Ellinas et al. challenged this notion. The authors showed that BMI was not an independent predictor of difficulty in 427 patients [37]. On the other hand, Stiffler et al. reported that palpation to identify lumbar spine bone landmarks was difficult in 68% of obese patients compared to 5% in their non-obese counterparts [38]. We did not evaluate the degree of difficulty to perform neuraxial blocks in our study; however, we did not find differences in the rate of failed neuraxial blocks leading to transition to general anesthesia in obese patients. Finally, morbid obesity has been associated with more frequent persistent systolic and diastolic hypotension in direct relationship with increasing BMI [39]. We did not assess the effect of anesthetic techniques on hemodynamic variables; however, since the most common strategy to treat hypotension during cesarean section under neuraxial anesthesia is the administration of intravenous phenylephrine, we consider that the total dose of this medication is a surrogate for hypotension. By using this surrogate, we did not found differences attributable to BMI.

Our results showed a significant inverse relationship between substance abuse and BMI. Sansone et al. reported a similar relationship in non-pregnant patients. The authors attribute the finding to a “brain reward site competition” among substances such as food and drugs [40]. This theory might explain the increased incidence of substance abuse among patients with obesity after bariatric surgery [41]. We think that our finding warrants further research as substance abuse during pregnancy is a public health problem with devastating consequences to mother and fetus [42]. Furthermore, future research should focus on specific types of eating behavior in relation to substance abuse and on the use of specific drugs [43].

This study has several limitations. We did not evaluate the level of difficulty associated with the insertion of epidural catheters and spinal anesthesia. Due to the retrospective nature of our study, information about difficult procedures was not recorded systematically; however, we assume that BMI was not a factor related to difficulty, as we were unable to demonstrate an increased rate of failed block or conversion to general anesthesia because of inability to perform the neuraxial procedure. Our study was not powered to detect differences in neonatal outcomes or postoperative obstetric adverse outcomes such as infection and wound complications.

In conclusion, our study shows that increasing BMI is associated with comorbidities such as hypertension and diabetes mellitus, as well as with increased intraoperative blood loss. We were unable to detect differences in other obstetric, anesthetic and neonatal outcomes.

## Provenance and peer review

Not commissioned, externally peer reviewed.

## Ethical approval

IRB approval (Augusta University). Project number: **1053583**–**4**.

## Funding

Funded by the authors.

## Conflicts of interest

None.

## Author contribution

Efrain Riveros-Perez: Data collection and analysis. Manuscript construction.

Alexander Rocuts: Data collection and analysis. Manuscript review.

Jennifer Xiong: Data collection. Manuscript construction.

Jacob McClendon: Data collection. Manuscript construction.

Thomas Cheriyan: Data collection analysis. Manuscript construction.

## Research registration number

The study is registered with ISRCTN registry (registration number: ISRCTN 16386326) and.

Clinicaltrials.gov. ID number NCT03590951.

## Guarantor

Efrain Riveros-Perez.

## Appendix A Supplementary data

Data profile Data profile
